# GROWTH DIFFERENTIATION FACTOR 15 IS CORRELATED TO MARKERS OF IMMUNOSENESCENCE IN MONOCYTES

**DOI:** 10.1093/geroni/igz038.387

**Published:** 2019-11-08

**Authors:** Brandt Pence, Johnathan Yarbro

**Affiliations:** University of Memphis, Memphis, Tennessee, United States

## Abstract

Immunosenescence is an age-associated decrease in function of immune cells precipitated by a variety of mechanisms and affecting nearly every immune cell subset. In myeloid cell subsets, aging reduces numbers of phagocytes and impairs their functional abilities, including antigen presentation, phagocytosis, and bacterial clearance. Recently, we have described an aging effect on several functions indicating immunosenescence in monocytes, including impaired mitochondrial function and reduced inflammatory cytokine gene expression during stimulation with lipopolysaccharide (LPS). We hypothesized that circulating factors altered by the aging process underly these changes. Growth/differentiation factor-15 (GDF-15) is a distant member of the transforming growth factor-beta superfamily that has known anti-inflammatory effects in macrophages and has recently been shown to be highly differentially expressed during aging. We used biobanked serum and plasma samples to assay circulating GDF-15 levels in subjects from our previous studies and examined correlations between GDF-15 levels and monocyte mitochondrial function and inflammatory responses. Monocyte interleukin-6 production due to LPS stimulation was negatively correlated to plasma GDF-15 levels (p = 0.046). Additionally, serum GDF-15 was positively correlated to circulating CD16+ monocyte proportions (p = 0.021) and negatively correlated to monocyte mitochondrial respiratory capacity (p < 0.001). Therefore, our data suggest that GDF-15 is a potential circulating factor affecting a variety of monocyte functions and promoting monocyte immunosenescence, and thus is an attractive candidate for therapeutic intervention to ameliorate this.

